# Mutation profiling in chinese patients with metastatic colorectal cancer and its correlation with clinicopathological features and anti-EGFR treatment response

**DOI:** 10.18632/oncotarget.8541

**Published:** 2016-04-01

**Authors:** Zhe-Zhen Li, Feng Wang, Zi-Chen Zhang, Fang Wang, Qi Zhao, Dong-Sheng Zhang, Feng-Hua Wang, Zhi-Qiang Wang, Hui-Yan Luo, Ming-Ming He, De-Shen Wang, Ying Jin, Chao Ren, Miao-Zhen Qiu, Jian Ren, Zhi-Zhong Pan, Yu-Hong Li, Jiao-Yong Shao, Rui-Hua Xu

**Affiliations:** ^1^ Department of Medical Oncology, Sun Yat-sen University Cancer Center, State Key Laboratory of Oncology in South China, Collaborative Innovation Center for Cancer Medicine, Guangzhou, P. R. China; ^2^ Department of Molecular Diagnostics, Sun Yat-sen University Cancer Center, State Key Laboratory of Oncology in South China, Collaborative Innovation Center for Cancer Medicine, Guangzhou, P. R. China; ^3^ State Key Laboratory of Biocontrol, School of Life Sciences, Sun Yat-sen University, Guangzhou, P. R. China; ^4^ Department of Colorectal Surgery, Sun Yat-sen University Cancer Center, State Key Laboratory of Oncology in South China, Collaborative Innovation Center for Cancer Medicine, Guangzhou, P. R. China

**Keywords:** colorectal cancer, mutation profile, RAS mutations, OncoCarta™ Panel, Chinese

## Abstract

An increasing number of studies reveal the significance of genetic markers in guiding target treatment and refining prognosis. This retrospective observational study aims to assess the mutation profile of metastatic colorectal cancer (mCRC) in Chinese population with the help of MassARRAY^®^ technique platform and OncoCarta™ Panel.

322 Chinese patients with mCRC who received clinical molecular testing as part of their standard care were investigated. 80 patients received cetuximab palliative treatment. 238 common hot-spot mutations of 19 cancer related genes in the OncoCarta™ Panel were tested.

44 mutations in 11 genes were detected in 156 cases (48.4%). At least one mutation was identified in 38.5% (124/322) of all tested cases, two concomitant mutations in 9.0% (29/322) and three mutations in 3 cases (<1%). *KRAS* was the most frequently mutated gene (34.8%), followed by *PIK3CA* (9.6%), *NRAS* (4.3%), *BRAF* (3.4%), *EGFR* (2.5%) and *HRAS* (1.2%). Less frequent mutations were detected in *PDGFRA*, *RET*, *AKT1*, *FGFR1*, and *ERBB2*. Co-mutation of RAS family subtypes was observed in 5 patients, and *KRAS* and *BRAF* concurrent mutation in 1 patient. *KRAS, NRAS, BRAF* and *PIK3CA* mutations had association with some clinicopathological features statistically. Patients identified as wild-type in all 19 genes had better objective response rate when treated with cetuximab.

The clinical molecular testing with OncoCarta™ Panel supplemented the limited data of mCRC in Chinese population, and offered a clearer landscape of multiple gene mutational profile in not only clinically prognostic *KRAS, NRAS, BRAF* and *PIK3CA* genes, but also less frequent mutated genes. Knowledge of these multiple gene mutation patterns may give clues in exploring interesting accompanying co-occurrence relationship or mutually exclusive relationship between mutated genes, as well as in predicting benefit of all-wild-type patients from anti-EGFR treatment.

## INTRODUCTION

Colorectal cancer (CRC) is the most common gastrointestinal malignancy occurring fourth in males and third in females across the globe, accounting for over 1.2 million new cases and 0.6 million deaths per year [1]. A good deal of researches were carried out in the molecular pathogenesis of CRC, discovering that activation of multiple signaling pathways plays an important role in regulating cell proliferation, angiogenesis, cell motility, and apoptosis [2, 3]. *KRAS, NRAS* and *BRAF* are the downstream oncogenes and their mutation may lead to activation of mitogen-activated protein kinase (MARK) pathway independent of the function of upstream epidermal growth factor receptor (EGFR) [4–6]. Clinically, their mutations are important predictive and prognostic markers when determining candidacy of anti-EGFR treatment [7–9]. Besides MARK pathway, another important signal pathway is the phosphatidylinositol-3-OH (PI3K) pathway, often activated by mutation in *PIK3CA* gene [3, 10, 11]. *PIK3CA* is also considered as a predictive and prognostic marker toward anti-EGFR therapy [12, 13]. Lots of reports have documented *KRAS, BRAF* and *PIK3CA* mutation frequency in CRC [14–16]. Increasing evidence revealed the usefulness of a full molecular profile in making treatment strategy for CRC patients. The genome-scale analysis of 276 cases from the Cancer Genome Atlas (TCGA) in 2012 demonstrated a few frequently occurred genes [17]. At the same time, many more mutations that are much less frequent are also detected in many different genes [15, 18–23]. Those infrequent mutated gene might have a synergic or independent effect with mutations in *KRAS, BRAF, NRAS* and *PIK3CA*, though the clinical value of most of them still remains to be uncovered.

There have been researches regarding the population-based differences in the clinicopathological features and the genetic profile of the same cancer, as well as the response to anticancer treatment. For instance, for lung adenocarcinoma, the Northeast Asian population has a higher prevalence of activating mutation of the EGFR tyrosine kinase domain [24]. In Chinese CRC population, there are data regarding the prevalence and clinical significance of *KRAS, BRAF*, *NRAS* and *PIK3CA* mutations [25, 26]. But for those less frequently mutated genes whose significance is yet to be discovered, published data are quite limited among Chinese population.

The Sequenom platform has developed MassARRAY^®^ gene profiling technique. It's based on a matrix-assisted laser desorption ionization–time of flight mass spectrometry (MALDI-TOF MS) to detect multiple gene mutations with high sensitivity and accuracy [27]. The OncoCarta™ panel is a set of pre-designed and pre-validated assays by the parallel analysis of 238 possible mutations in 19 clinically relevant genes with as little as 500 ng DNA per sample, including frequent mutated genes such as *KRAS, NRAS, BRAF* and *PIK3CA*, which are most clinically relevant for CRC. In addition, it also contains assays for other infrequent mutations in genes, such as *AKT1, EGFR, HRAS, NRAS, MET* and others. Our center has been performing clinical molecular testing with OncoCarta™ Panel on metastatic colorectal cancer (mCRC) patients since 2014. This testing was performed on the group of mCRC patients for whom testing result would assist in identifying targeted therapies according to genotype pattern. We conducted this retrospective study to investigate the genetic profile in Chinese population, as well as to investigate the relationship between mutational status and the clinicopathological features. In addition, this study also explored the correlation between mutational profile and anti-EGFR treatment response.

## RESULTS

### Main patient characteristics

322 Chinese patients with metastatic colorectal cancer were considered eligible. Among the detected samples, 270 (83.9%) samples were from primary tumors, 38 (11.8%) from metastatic sites and the rest 14 (4.35%) were unknown. The main metastatic sites included liver in 188 (58.4%) patients, lung in 101 (31.4%), distant lymph node in 121 (37.6%), peritoneum in 95 (29.5%), and bone in 32 (9.9%). Other metastasis included uterus, ovary, adrenal gland, spleen, skeletal muscle and so on. Main patient characteristics are listed in Table 1.

**Table 1:** Main characteristics of 322 patients with metastatic colorectal cancer and the association of mutation profile with clinicopathological parameters

Clinicopathological features	Total samples, N=322	n (%)	*KRAS*		*NRAS*		*BRAF*		*PIK3CA*		Mutation numbers		
			Mutation (%)	*P*	Mutations (%)	*P*	Mutations (%)	*P*	Mutations (%)	*P*	Single mutations (%)	≥2 mutations (%)	*P*
Sex													
	Male	195 (60.6)	64 (32.8)	.40	9 (4.6)	>.99	4 (2.1)	.12	17 (8.7)	.56	176 (90.3)	19 (9.7)	>.99
	Female	127 (39.4)	48 (37.8)		5 (3.9)		7 (5.5)		14 (11.0)		114 (89.8)	13 (10.2)	
Age													
	>60	95 (29.5)	30 (31.6)	.52	8 (9.4)	**.03**	2 (2.1)	.52	12 (12.6)	.30	84 (88.4)	11 (11.6)	.54
	≤60	227 (70.5)	82 (36.1)		6 (2.6)		9 (4.0)		19 (8.4)		206 (90.7)	21 (9.3)	
	Median	52											
	Range	15-82											
Tumor differentiation													
	Well/Moderate	213 (66.1)	84 (39.4)	**.02**	9 (4.2)	>.99	8 (3.8)	.76	26 (12.0)	**.03**	186 (87.3)	27 (12.7)	**.03**
	Poor	109 (33.9)	28 (25.7)		5 (4.6)		3 (2.8)		5 (4.6)		104 (95.4)	5 (4.6)	
Tumor type													
	Papillary/tubular adenocarcinoma	288 (89.4)	104 (36.1)	.18	14 (4.9)	.38	10 (3.5)	>.99	31 (10.8)	.06	256 (88.9)	32 (11.1)	**.03**
	Mucinous/signet ring cell	34 (10.6)	8 (23.5)		0 (0.0)		1 (2.9)		0 (0.0)		34 (100)	0 (0.0)	
Primary tumor site													
	Right colon	84 (26.1)	36 (42.8)	.20	4 (4.8)	.91	4 (4.8)	.79	14 (16.7)	**.04**	73 (86.9)	11 (13.1)	.53
	Left colon	127 (39.4)	40 (31.5)		5 (3.9)		5 (3.9)		8 (6.3)		115 (90.6)	12 (9.4)	
	Rectum	84 (26.1)	28 (33.3)		4 (4.8)		2 (2.4)		6 (7.1)		77 (91.7)	7 (8.3)	
	Multiple origin	9 (2.8)	5 (55.6)		0 (0.0)		0 (0.0)		0 (0.0)		9 (100)	0 (0.0)	
	Missing	18 (5.6)											
Family history													
	With family history	92 (28.6)	35 (38.0)	.60	5 (5.4)	.76	6 (6.5)	.11	10 (10.9)	.53	83 (90.2)	9 (9.8)	>.99
	No family history	196 (60.9)	68 (34.7)		8 (4.08)		5 (2.6)		17 (8.7)		175 (89.3)	21 (10.7)	
	Missing	34 (10.6)											
Metastasis	Liver	188 (58.4)	72 (38.3)	.25	8 (4.2)	.77	5 (2.6)	.20	19 (10.1)	.67	169 (89.9)	19 (10.1)	.84
	Lung	101 (31.4)	41 (40.6)	.25	4 (4.0)	>.99	3 (3.0)	.75	9 (8.9)	>.99	89 (88.1)	12 (11.9)	.55
	Distant Lymph nodes	121 (37.6)	35 (28.9)	**.05**	5 (4.1)	>.99	7 (5.8)	.21	9 (7.4)	.42	109 (90.1)	12 (9.9)	>.99
	Peritoneum	95 (29.5)	36 (37.9)	.70	2 (2.1)	.23	8 (8.4)	**.007**	9 (9.5)	>.99	85 (89.5)	10 (10.5)	>.99
	Bone	32 (9.9)	11 (34.3)	>.99	0 (0.0)	.37	3 (9.4)	.11	4 (12.5)	.52	28 (87.5)	4 (12.5)	.76

Bold figures represent *P*<0.05.

Of these 322 patients, 80 (19.6%) patients received anti-EGFR treatment. Cetuximab was administrated as single agent or in combination with 5-FU/oxaliplatin/irinotecan regimen in palliative treatment. As first-line treatment 51 (63.8%) patients received cetuximab and second-line in 14 (17.5%). 15 (18.8%) patients were treated with cetuximab in third-line or beyond. No patient received panitumumab treatment.

### Mutational profile

**Figure 1:**
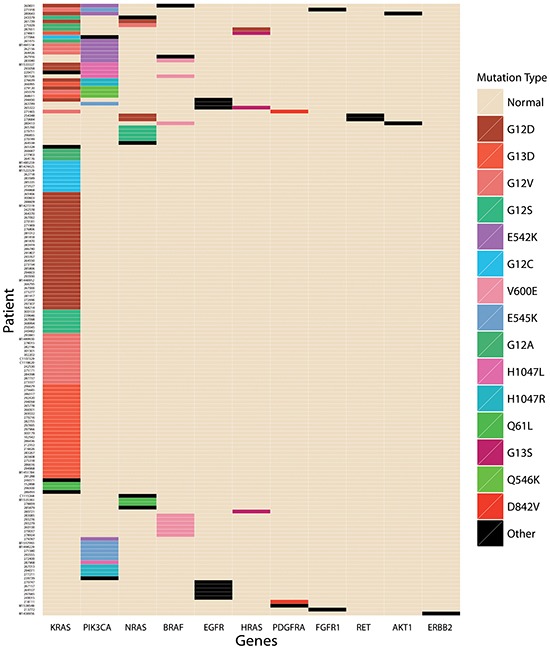
A schematic map of mutated genes in the 156 patients with at least one mutation in any of the 19 genes

Out of 322 tumors, 166 (51.6%) were all wild-type, defined as no mutation in any of the 19 genes listed in Table 2. At least one mutation was identified in 156 (48.4%) cases. In total, there were 44 mutations in 11 genes detected in the OncoCarta™ Panel, in 156 cases (Table 3). *KRAS* was the most commonly gene (112; 34.8%), followed by *PIK3CA* (31, 9.6%) *NRAS* (14, 4.3%) and *BRAF* (11, 3.4%). No mutation was identified in *ABL1, AKT2, CDK, FGFR3, FLT3, JAK, KIT* or *MET*. One single mutation was present in 38.5% (124/322) of all tested cases, two concomitant mutations in 9.0% (29/322) and three mutations in 3 tumors (<1%). A schematic map of the 156 patients with at least one mutation in any of the 19 genes is shown in Figure 1.

**Table 2:** Genes included in the OncoCarta panel

Panel of genes analyzed		
*ABL1*	*FGFR1*	*JAK*
*AKT1*	*FGFR3*	*KIT*
*AKT2*	*FLT3*	*MET*
*BRAF*	*KRAS*	*PDGFRA*
*CDK*	*NRAS*	*PIK3CA*
*EGFR*	*HRAS*	*RET*
**ERBB2**		

**Table 3:** Summary of mutations identifies in 322 patients with metastatic colorectal cancer

	Mutation		N% (322)
Gene	Mutation	Cases	
*KRAS*		**112**	**34.8**
*NRAS*		**14**	**4.3**
*HRAS*		**4**	**1.2**
*BRAF*	V600E	9	
	G464E	1	
	G469A	1	
	**total**	**11**	**3.4**
*PIK3CA*	C420R	1	
	E542K	9	
	E545K	7	
	H1047L	5	
	H1047R	5	
	M1043I	1	
	Q546K	3	
	**total**	**31**	**9.6**
*EGFR*	L747-P753>S	1	
	G719S	1	
	P772-H773insV	1	
	E709K	1	
	H773-V774insNPH	2	
	P753S	1	
	D770-N771insG	1	
	**total**	**8**	**2.5**
*PDGFRA*	D842V	2	
	T674I	1	
	**total**	**3**	**0.9**
*FGFR1*	S125L	1	
	I836del	1	
	**total**	**2**	**0.6**
*RET*	C643Y	**2**	**0.6**
*AKT1*	E17K	**2**	**0.6**
*ERBB2*	G776S	**1**	**0.3**

**Figure 2:**
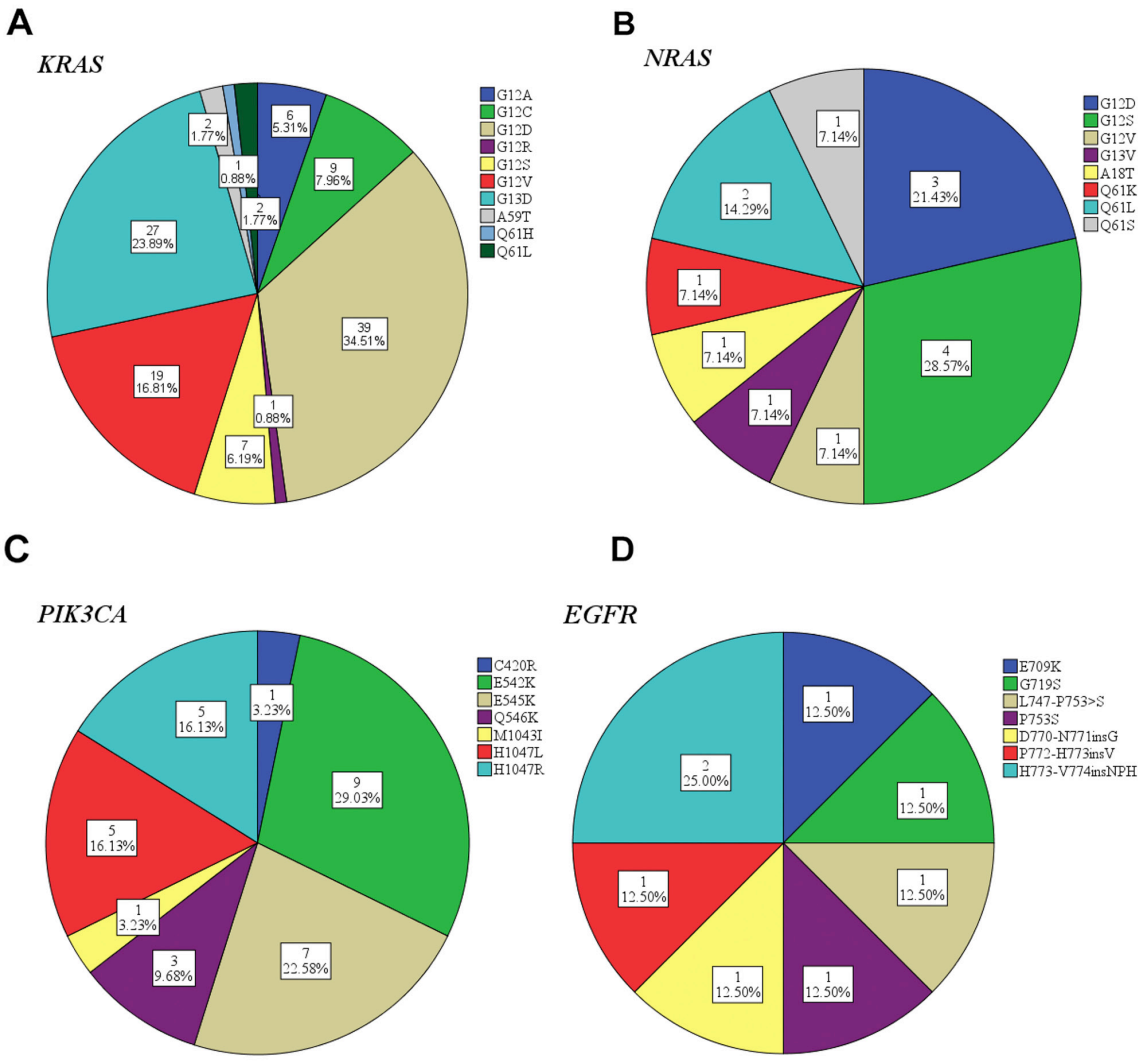
Mutation subtypes frequency distribution of *KRAS* A. *NRAS* B. *PIK3CA* C. and *EGFR* D

The family members of human *RAS* genes include *HRAS, KRAS* and *NRAS* genes. At least one gene mutation of the *RAS* family was identified in 125 (38.8%) tumors (details shown in Table 4). The most frequent mutation occurred in codon 12 for both *KRAS* and *NRAS*. One patient harbored a double *KRAS* mutation in both codon 12 and codon 59 (G12D, A59T). The distribution of mutation subtypes is summarized in Figure 2. Unlike the *KRAS* and *NRAS* genes, the status of *HRAS* mutation was detected in only 4 (1.2%) cases. Among them, G13S mutation in codon 13 was identified in 3 tumors, and G12D mutation in codon 12 in 1 case.

**Table 4:** Frequency of mutation in *RAS* family in patients with metastatic colorectal cancer

Genes		Cases with mutation (%)
Total cases with *RAS* mutation		125(38.8)
Total cases with *KRAS* mutation		112 (34.8)
*KRAS* codon 12	G12A	6 (1.9)
	G12C	9 (2.8)
	G12D	39 (12.1)
	G12R	1 (0.3)
	G12S	7 (2.2)
	G12V	19 (5.9)
*KRAS* codon 13	G13D	27 (8.4)
*KRAS* codon 59	A59T	2 (0.6)
*KRAS* codon 61	Q61H	1 (0.3)
	Q61L	2 (0.6)
Total cases with *NRAS* mutation		14 (4.4)
*NRAS* codon 12	G12D	3 (0.9)
	G12S	4 (1.2)
	G12V	1 (0.3)
*NRAS* codon 13	G13V	1 (0.3)
*NRAS* codon 18	A18T	1 (0.3)
*NRAS* codon 61	Q61K	1 (0.3)
	Q61L	2 (0.6)
	Q61S	1 (0.6)
Total cases with *HRAS* mutation		4 (1.2)
*HRAS*	G12D	1 (0.3)
	G13S	3 (0.9)

Notably, in our study we identified 5 cases in which the different genes mutation in *RAS* family concomitantly existed. There were 3 patients detected with *KRAS* and *NRAS* concurrent mutation. The exact *KRAS* and *NRAS* mutation was G12A and G12V, G12D and G12D, G12S and A18T respectively for the 3 cases (Figure 1). In addition, 2 patients were detected with concurrent *KRAS* and *HRAS* mutation. One of them was identified *KRAS* G12A mutation and *HRAS* G12D mutation, and the other exhibited G13D for *KRAS* and G13S for *HRAS*. No concurrent *NRAS* and *HRAS* mutation was detected in our study. These findings reveal that the mutations in RAS family proto-oncogene are not mutually exclusive.

*BRAF* gene, another important incidence on EGFR pathway, was found to be mutant in exon 15 (9 cases) and exon 11 (2 cases), with V600E mutation as the most frequent subtype. In our study group, there was 1 patient detected with concurrent *KRAS* G12D mutation and *BRAF* G464E mutation, together with *PIK3CA* E452K mutation. However, we found no co-mutation of *BRAF* V600E spot with any *KRAS*.

The frequency of *PIK3CA* mutation is the second highest (31/322, 9.6%), following *KRAS* mutation. Mutations in exon 9 coding for the helical domain (C420R, E542K, E545K and Q546K) were found in 20 patients. Exon 20 mutations coding for the kinase domain (H1047L, H1047R and M1043I) were found in 11 patients (shown in Figure 2). Mutations in *PIK3CA* tended to be accompanied with a second or even a third mutation. Notably, *PIK3CA* exon 9 mutation were significantly associated with *KRAS* mutation (11/112 [9.8%] vs 9/210 [4.3%] *P*=.04), whereas *PIK3CA* exon 20 didn't show such an association (5/112 [4.5%] vs 6/210 [2.9%], *P*=.52). As for *PIK3CA* exon 9 and *BRAF* co-mutation, this significant association also existed (3/11 [27.3%] in *BRAF* mutant vs 17/311 [5.5%] in *BRAF* wild-type, *P*=0.03), while no significant correlation existed between *PIK3CA* exon 20 and *BRAF* (1/11 [9.1%] in *BRAF* mutant vs 10/311 [0.3%] in *BRAF* wild-type, *P*=.32), thereby suggesting that *PIK3CA* exon 9 may occur as a second mutation in the later stage in the carcinogenesis.

**Figure 3:**
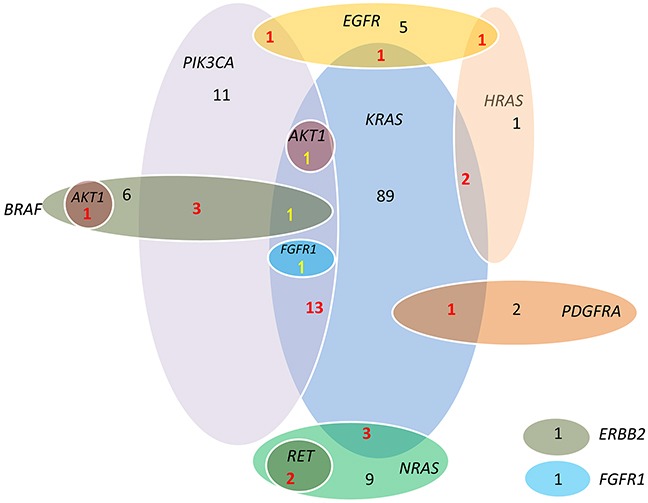
Associations among *KRAS, NRAS, HRAS, BRAF, PIK3CA, EGFR, FGFR1, PDGFRA, RET, AKT1* and *ERBB2* mutations Mutations of three genes from *RAS* family are not mutually exclusive, neither were the *KRAS* and *BRAF*. *RET* mutations co-occurred with *NRAS* mutation. *AKT1* mutation co-occurred with *RAS/RAF*.

In our study, less frequent mutations in CRC were also found in *EGFR* (8), *PDGFRA* (3), *RET* (2), *AKT1* (2), *FGFR1* (2), and *ERBB2* (1). Some of these genes were presented as single mutation, while some co-occurred with *KRAS, NRAS, BRAF* and *PIK3CA*. 3 of the 8 *EGFR* mutation (Figure 2) were accompanies with *KRAS, HRAS* and *PIK3CA* mutations, all of which exist in the downstream signal pathways of *EGFR*. Interestingly, there were 2 cases identified with *RET* C643Y mutation, and there seemed to be a strong correlation with *NRAS* mutation (2/14 [14.3%] in *NRAS* mutant vs 0/308 [0%] in *NRAS* wild-type, *P*=.002). Both cases identified with *AKT1* mutation had concomitant *RAS/RAF* mutation, one with *BRAF* V600E mutation, the other with *KRAS* codon 12 and *PIK3CA* exon 9 mutations. Only one *ERBB2* mutation was detected, presenting a frequency of 0.3%. The concomitant and exclusive relationship among all 11 genes is visualized in Figure 3.

### Exploratory analysis of mutation profile and clinicopathological characteristics

An exploratory analysis of the clinicopathological characteristics by genetic profile was performed. *KRAS* mutation rate was higher in patients with histologically well/moderate grade tumor (39.4% vs 25.7%, *P*=.02) or in patients without distant lymph node metastasis (40.8% vs 28.9%, *P*=.05). *NRAS* showed a higher mutation frequency in patients older than 60 years (9.4% vs 2.6%, *P*=.03). *BRAF* mutation trended toward an association with peritoneum implantation (8.4% vs 1.5%, *P*=.007). *PIK3CA* mutation frequency was higher in well/moderate differentiation tumors (12.0% vs 4.6%, *P*=.03), and right colon as the primary tumor site (*P*=.04). They trended toward a positive association with papillary or tubular adenocarcinoma (10.8% vs 0.0%, *P*=.06). Patients with more than one mutation in any of the genes detected in OncoCarta™ Panel were associated with better differentiation (12.7% vs 4.6%, *P*=.03) and papillary or tubular adenocarcinoma (11.1% vs 0.0%, *P*= .03). Mutation profile and clinical correlations are summarized in Table 1.

### Anti-EGFR treatment response by mutation profile

Among the 80 patients treated with cetuximab, the objective response was a CR in 1 (1.3%) patient, PR in 30 (37.5%), stable disease (SD) in 18 (22.5%) and progression disease (PD) in 14 (17.5%). In 17 patients, the objective response couldn't be evaluated, either because the medical documentation was incomplete or the patients were initiating cetuximab treatment at the time when the information was collected. All those 80 patients were *RAS* wild-type. In our analysis, better objective response rate (ORR) was strongly correlated with patients who presented wild-type in all the 19 genes (31/56 [55.3%] in all wild-type vs. 0/7 [0%] in any mutation, *P*=.006). During a relatively short follow-up time (median 8.2 months, range 2.2-42.0 months), survival information was collected successfully collected in only 15 patients. However in the survival analysis, the small group sample size did not present any significant result between mutation profile and survival benefit (overall survival and progression-free survival), including mutation number analysis (data not shown).

## DISCUSSION

During the past decades, molecular testing has been attached greater and greater significance with the progress in targeted treatment. In colorectal cancer, data have been accumulated in genetic profiling [15, 18–20, 28, 29]. In this cohort of 322 mCRC patients, our study described a genotype distribution picture among Chinese population, with an accurate and sensitive multiple gene detection technique on MassARRAY^®^ platform.

Among the 19 genes tested in the OncoCarta™ Panel, *KRAS* and *NRAS* belong to the most clinically relevant genes in CRC. In our current study, the frequency of *KRAS* mutations was 34.8%, consistent with most reports from other populations [29–32], suggesting that such an alternation exists in 30-40% of colorectal cancer. Compared to the Catalogue of Somatic Mutations in Cancer (COSMIC) database and other publication [33], the frequency and specific amino acid mutations detected here were similar to western countries [34]. However, the correlation between *KRAS* and clinicopathological parameters remains controversial according to different reports. Our finding suggested that *KRAS* mutation is more frequent in well and moderately differentiated tumors than poorly differentiated ones. This finding conflicted with some publications in which the *KRAS* mutation is associated with older age, deeper invasion, and poorer differentiation [35, 36]. The difference may be explained by the distinction of selected patients and sample sources. In our study, only metastatic patients were included and the tested samples came from both surgery and biopsy, both primary tumor and metastasis, both pre-treatment and post-treatment stage. More reliable data are needed to confirm the *KRAS* correlation with clinicopathological features. The prevalence of *NRAS* mutation in our study is 4.4%, in accordance with reported range in literature between 2.6% and 7% [8, 37–39]. We observed that *NRAS* mutations were more common in patients older than 60 years, similar to previous publications [20]. In melanomas and leukemia, this association was also discovered [40]. Unlike *KRAS* and *NRAS* from the same family, the clinical significance of *HRAS* mutation has not been convincingly elucidated so far. We detected a *HRAS* mutation frequency of 1.2%, slightly higher than the estimated frequency of lower than 1 % in a previous review [41]. No significant correlation with clinicopathological features was identified probably due to the limited number of *HRAS* mutation. Some observations deserve further discussion. First of all, one patient with both *KRAS* codon 12 and codon 59 mutations caught our attention, indicating that concurrent mutations may exist in different *KRAS* exons. This suggests heterogeneity may occur in one single tumor lesion. Secondly, to our knowledge, this is the first report showing that *KRAS*, *NRAS* and *HRAS* mutations are not mutually exclusive. This phenomenon conflicted with several previous reported suggesting mutually exclusive mutations in *RAS* family [8, 35]. One possible explanation may be the low detection rate of *KRAS* exon3 and 4, *NRAS* and *HRAS* mutation limited the discovery of potential co-occurrence. Hopefully with accumulated data in multiple gene profile, relationship among *RAS* family members may be better elucidated.

We identified 11 *BRAF* mutations among 322 samples. As a poor prognostic mutation recognized widely, lots of researches reported the associations between *BRAF* mutation with primary tumor site, age, gender and differentiation grade [35, 42, 43]. But in our study we didn't find the association, possibly because of the bias resulted from the limited number of detected mutations in our sample. Yet the higher prevalence in patients with peritoneum implantation somehow indicated the poor predictive value. As for the relationship between *KRAS* and *BRAF* mutations, we identified a patient with concomitant *KRAS* G12D mutation and *BRAF* G464E mutation. This test result shared a similar phenomenon in a previous report: though *KRAS* and *BRAF* V600E mutations are mutually exclusive, it is possible that *BRAF* non-V600E mutation can co-exist with *KRAS* mutation in the same case; this condition is rarely observed mainly because of the low frequency of BRAF non-V600E mutation [44].

Some previously published studies have suggested that *PIK3CA* mutations were associated with *KRAS* mutation [8, 25, 45]. In agreement with the conclusion from a large retrospective European study that analyzed 1022 CRC samples [8], we detected a strong association between exon 9 *PIK3CA* and *KRAS/BRAF* mutations. As expected, this association didn't apply to *PIK3CA* exon 20. This can be putatively explained by the research finding that *PIK3CA* exon 9 mutations (coding for helical domain) adjusted function though a Ras-GTP dependent pattern, whereas the gain of function induced by *PIK3CA* exon 20 mutations (coding for kinase domain) didn't require the involvement of Ras [46].

In our study, we also identified 6 more genes in CRC involved different signaling pathways, including RAS/MAPK and PI3K/AKT pathway. Previous reports about these gene mutations are quite limited in CRC. *EGFR*, an upstream element in signaling pathway, is a significant predictive and prognostic indicator in lung adenocarcinoma to guide the decision of targeted treatment. In our study of CRC, 8 *EGFR* mutations were detected, with frequency of 2.5%. This prevalence is much lower than that in lung adenocarcinoma in Asian populations, about 30% [24]. *EGFR* mutations are not exclusive with downstream mutations according to our study. *PDGFRA* mutations are often studied in gastrointestinal stromal tumor (GIST) [47]. To our knowledge, this is the first report of *PDGFRA* mutations in CRC, with a frequency of 0.9%. Preclinical researches have elucidated the role of *FGFR1* in regulating CRC cell behavior and *FGFR1* is considered a putative therapeutic target in early phase trials [48, 49]. Previous reports showed *FGFR1* gene amplification rate was 3.8% in 291 CRC cases [50], whereas our study suggested a 0.9% *FGFR1* mutation rate. Oncogenic *RET* point mutations and *RET* fusions occur mainly in papillary thyroid cancer and lung adenocarcinoma [51]. The *RET* gene mutation frequency was 0.6% in our study. To be noted, all the 2 *RET* mutations had concurrent *NRAS* mutation. This phenomenon has not been reported before, deserving more exploration in the correlation of *NRAS* and *RET* mutation. *AKT1* is an important component in PI3K/AKT/mTOR pathway. Our findings were quite similar to the conclusion of a previous study that *AKT1* frequency was 0.7% in CRC and tended to co-occur with *RAS/RAF*-activating mutation [21]. The detection of *ERBB2* mutation was also reported in TCGA [17] and these patients may benefit from target treatment with *ERBB2* antibody. Although clinical significance of these infrequent genes is not yet uncovered, the occurrence of such mutation gives us more insight into the complexity of cancer cell genotype and offers more clues as treatment target.

Since anti-EGFR monoclonal antibody became an effective treatment, mutation detection of *KRAS* and *NRA*S has been recommended by the National Comprehensive Cancer Network (NCCN) and the European Society for Medical Oncology (ESMO) guidelines to avoid the inefficacy in *RAS* mutant groups. However, up to 65% of patients with *KRAS* wild-type tumors are resistant to anti-EGFR monoclonal antibodies. Our finding suggested that the treatment response of cetuximab was more obvious in patients who presented wild-type in all the 19 genes than those who harbored at least one mutation in any genes. According to an European Consortium report, the all-wide-type patients (*KRAS, BRAF, NRAS* and *PIK3CA*-exon 20 wide-type) reached the highest ORR from anti-EGFR therapy in chemo-refractory setting. Our finding supplemented this conclusion, with expansion to more infrequent genes in addition to *KRAS, BRAF, NRAS* and *PIK3CA*, and expansion to all cetuximab settings other than chemo-refractory condition. This conclusion suggests that in order to improve the ORR, multiple gene detection can be recommended to decide the all wide-type patients. Larger samples are needed to further confirm the predictive value of multiple mutation profiling.

There are several limitations in our study that merits further discussion. This study is retrospective and exploratory in nature and the drawing of more convincing conclusion is thus limited. Due to the restrictions of medical record documentation and short follow-up time, we failed to collect adequate treatment and survival information in anti-EGFR treated patients. In addition, the OncoCarta™ panel based on MassARRAY^®^ platform identified 19 genes that were predetermined with a bias toward detecting hot-spot mutations which may be considered for targeted treatment. This may miss other potential mutations throughout the coding sequence.

In conclusion, we conducted a retrospective study to describe a Chinese mCRC mutational profile and performed exploratory analysis to make clinical correlations. These findings supplemented the limited data of mCRC in Chinese population, and offered us a clearer image of multiple gene mutational profile in not only clinically prognostic *KRAS, NRAS, BRAF* and *PIK3CA* gene, but also less frequent mutated genes such as *HRAS, EGFR, PDGFRA*, *RET*, *AKT1*, *FGFR1*, and *ERBB2*. Knowledge of these gene mutation patterns may give clues in exploring interesting accompanying co-occurrence relationship or mutually exclusive relationship between mutated genes. It may also help to predict benefit of all-wild-type patients from anti-EGFR treatment. Hopefully the genotype landscape will advocate the development of precision medicine.

## MATERIALS AND METHODS

### Study population and clinical data

After approval from the local Institutional Review Board, we retrospective investigated 322 mCRC patients who received clinical molecular testing as part of their standard care at Sun Yat-sen University Cancer Center (Guangzhou, China) between August 2014 and July 2015. Patients were chosen to undergo testing at the discretion of their treating physician. Principal inclusion criteria were as follows: histologically confirmed papillary/tubular adenocarcinoma, signet ring carcinoma and mucinous carcinoma of the colon or rectum; and presence of unresectable metastatic disease. Patient's nationality of China was also required to be included into this population-specific study. CRC diagnosis was confirmed by hematoxylin and eosin (HE) staining and histological analysis. Informed consent was obtained from all individual participants included in the study, giving their authorization to access their clinical information and tumor samples for research purpose.

Clinical data were retrieved by medical record archive, including age, sex, previous resection and site of primary tumor, number and type of received treatments, exposure to EGFR inhibitors or antiangiogenics, treatment response, type and date of metastasectomy. Pathologic data consisted of tumor size, tumor location and grade, histological type, lymphovascular and perineural invasion. Tumors were classified for histological type and grade using the current World Health Organization criteria by two independent pathologists. Since all patients included in this study were with unresectable metastasis considered as Stage IV, the TNM stage of each patient was omitted. Objective tumor response was evaluated every 6 weeks by computing tomography scan according to Response Evaluation Criteria in Solid Tumors (RECIST 1.1). Responders were defined as the patients who had a complete response (CR) or partial response (PR).

### Tissues and mutation detection

Each patient included in the analysis has provided available and adequate FFPF tumor specimens for molecular analysis. These specimens were resected at various cancer hospitals and sent to our center. Surgery primary CRC samples, resected metastasis or small biopsies samples were accepted. Routinely processed HE staining slides were reviewed by a pathologist to determine tumor adequacy and to select the area of highest tumor percentage. Sections (4–6μm) were cut and transferred to 1.5 mL Eppendorf tubes for DNA extraction. DNA was extracted using the QIAamp DNA FFPE Tissue Kit (Qiagen, Hilden, Germany), according to the manufacturer's protocol. The quantity and quality of the isolated DNA were tested using a Nanodrop ND-2000 Spectrophotometer (Thermo Scientific, Niederelbert, Germany). The final DNA samples were diluted to 10ng/μL for analysis.

For mutation detection, the OncoCarta Panel v. 1.0. (Sequenom Inc., San Diego, CA, USA) were used and the protocol provided by the manufacture was followed. As was described by Zhang [27], 20 ng of DNA was amplified using 24 sets of OncoCarta™ polymerase chain reaction (PCR) primers. An extension reaction based on the OncoCarta™ extension primers was then conducted. After salts were removed with a cation exchange resin, the products were spotted onto a 384-well SpectroChipII using the MassARRAY^®^ Nanodispenser RS1000 (Sequenom Inc.) and analyzed on a MALDI-TOF mass spectrometer (Sequenom Inc.). In each experiment, HPLC-purified water was selected as the blank control and normal human somatic cells as the negative control. The OncoCarta™ Panel has the capacity to detect 238 mutations in 19 genes, listed in Table 2. A successful experiment should satisfy the standard of that the sample figure was typical and the blank control had no peak. Preliminary analysis of mutation data was performed by the software MassARRAY TYPER 4.0 (Sequenom Inc., San Diego, USA). The results of mutation frequency with more than 5% integrated with medium or high credibility were re-analyzed, while those with 5-10% and medium credibility were validated using fluorescent qRT-PCT. The false-positive results generated by ion disturbance were excluded from the analysis.

### Statistical analysis

Since this analysis was exploratory, no sample size was calculated. Descriptive analysis for clinical and molecular data was performed. Statistical analysis was carried out by the IBM SPSS® Statistics 21.0.0 package software (SPSS Inc). Frequency distributions for categorical variables and mean with standard deviation, 25th and 75th percentiles for continuous variables were calculated. Pearson's Chi-square (χ^2^) test was used to compare the proportion of gene mutations among groups with different clinicopathologic variables. All the P values were two-tailed, and the statistical significance was set at *P*<0.05.

## Abbreviations

CRC: colorectal cancer
mCRC: metastatic colorectal cancer
MARK: mitogen-activated protein kinase
EGFR: epidermal growth factor receptor
PI3K: phosphatidylinositol-3-OH kinase
TCGA: the Cancer Genome Atlas
MALDI-TOF MS: matrix-assisted laser desorption ionization–time of flight mass spectrometry
FFPE tissue: formalin-fixed paraffin-embedded tissue
HE staining: hematoxylin and eosin staining
RECIST: Response Evaluation Criteria in Solid Tumors
CR: complete response
PR: partial response
SD: stable disease
PD: progression disease
PCR: polymerase chain reaction
ORR: objective response rate
COSMIC: Catalogue of Somatic Mutations in Cancer
GIST: gastrointestinal stromal tumor
NCCN: the National Comprehensive Cancer Network
ESMO: the European Society for Medical Oncology.
